# Displacement in root apex and changes in incisor inclination affect alveolar bone remodeling in adult bimaxillary protrusion patients: a retrospective study

**DOI:** 10.1186/s13005-020-00242-2

**Published:** 2020-11-20

**Authors:** Huimin Mao, Andi Yang, Yue Pan, Houxuan Li, Lang Lei

**Affiliations:** 1 Department of Orthodontics, Nanjing Stomatological Hospital, Medical School of Nanjing University, Nanjing, China; 2 Department of Periodontics, Nanjing Stomatological Hospital, Medical School of Nanjing University, Nanjing, China

**Keywords:** Bimaxillary protrusion, Alveolar bone remodeling, Orthodontic tooth movement, Periodontal

## Abstract

**Background:**

Periodontal health is of great concern for periodontists and orthodontists in the inter-disciplinary management of patients with bimaxillary protrusion. The aim of present study is to investigate changes in the alveolar bone in the maxillary incisor region and to explore its relationship with displacement of root apex as well as changes in the inclination of maxillary incisors during incisor retraction.

**Methods:**

Samples in this retrospective study consisted of 38 patients with bimaxillary protrusion. Cone-beam computed tomography (CBCT) images was taken before(T0) and after (T1) treatment. Alveolar bone thickness (ABT), height (ABH) and area (ABA) were utilized to evaluate changes in the alveolar bone, while incisor inclination and apex displacement were used to assess changes in the position of maxillary central and lateral incisors. Correlations between alveolar bone remodeling and apex displacement as well as changes in the inclination were investigated.

**Results:**

The labial ABT of central and lateral incisors at the mid-root third was increased. In contrast, the palatal ABT at crestal, mid-root and apical third level were consistently decreased. ABH was not altered on the labial side, while significantly decreased on the palatal side. ABA was not significantly increased on the labial side, but significantly decreased on the palatal side, leading to a significantly reduced total ABA. Orthodontic treatment significantly reduced inclination of upper incisors. Changes in the amount (T1-T0) of ABA was remarkably correlated with apex displacement and changes of inclination (T1-T0); in addition, using the multivariate linear regression analysis, changes of ABA on the palatal side (T1-T0) can be described by following equation: Changes of palatal ABA (T1-T0) = − 3.258- 0.139× changes of inclination (T1-T0) + 2.533 × apex displacement (T1-T0).

**Conclusions:**

Retraction of incisors in bimaxillary protrusion patients may compromise periodontal bone support on the palatal side. An equation that incorporated the displacement of root apex and change in the incisor inclination may enable periodontist-orthodontist interdisciplinary coordination in assessing treatment risks and developing an individualized treatment plan for adult patients with bimaxillary protrusion. Moreover, the equation in predicating area of alveolar bone may reduce the risks of placing the teeth out of the bone boundary during 3D digital setups.

## Background

Bimaxillary protrusion, a well-recognized malocclusion in Eastern Asian communities, is characterized by proclined incisors as well as protruded lips [1]. Negative perception of protrusive dentitions and lips is one major concern for patients to seek orthodontic care [2]. Four-premolar extraction, maximal anchorage and incisor retraction are often included in the treatment to achieve optimal improvement in the lip morphology [3]. Although dynamic remodeling of alveolar bone might ensue orthodontic tooth movement (OTM) [4–6], the large retraction of incisors poses a great challenge to the periodontal health in patients with bimaxillary protrusion. Precise treatment planning and risk assessment may help reduce the periodontal sequelae in patients with bimaxillary protrusion [7].

Despite the persistent controversy regarding the alveolar bone remodeling pattern during OTM, i.e. “through the bone” or “with the bone” theory [8], the 2014 American Association of Periodontal Regeneration World Workshop systematic review reports that the direction of the tooth movement and the bucco-lingual thickness of the gingiva play important roles in soft tissue alterations during orthodontic treatment [7]. Therefore, to reduce the periodontal risk in patients with bimaxillary protrusion, an inter-disciplinary approach involving the orthodontists and periodontists in the decision-making process would definitely reduce periodontal sequelae and improve orthodontic treatment outcome [9]. Anatomic features of local alveolar process, periodontal remodeling potential and orthodontic tooth movement pattern should be included in determining overall treatment plan and specific force system.

Cone-beam computed tomography (CBCT) images are being widely used in orthodontics to assess 3-dimensional (3D) spatial relationship in the diagnosis of impacted teeth [10]. In addition, 3D imaging allows for accurate detection of anatomic features of alveolus, which is superior to lateral cephalometric, intraoral periapical or panoramic radiographs [11]. Information on the alveolar bone surrounding incisors is of vital importance to determine the boundary of orthodontic tooth movement.

In addition to assessment of periodontal tissue condition and remodeling potential, accurate orthodontic treatment plan and precise tooth movement control should also be guaranteed to minimize periodontal risks. Upon application of orthodontic forces on the periodontal ligament, biological responses in the molecular and cellular level will lead to several types of OTM, including uncontrolled tipping, controlled tipping and bodily movement [12]. Such differed types of OTM may lead to varied displacement of the apex and incisor inclination although retraction of incisal edge is similar.

Orthodontic tooth movement is limited by the anatomic dentoalveolar boundaries that are set by the cortical plates of the alveolus at the level of the incisor apices [5]. Digital setup by available softwares, such as OrthoCAD (Align Technology, San Jose, Calif), SureSmile (Orametrix, Richardson, Tex), and Orchestrate (Orchestrate, Rialto, Calif), has been widely utilized to mimic tooth movement and predict final position of the dentition rather accurately [13]. However, without taking the alveolar anatomy and its remodeling capacity into consideration, digital setup-aided orthodontics as well as traditional orthodontics may possibly put the dentition out of the boundary, leading to instability, root resorption and alveolar bone loss [7].

Currently, CBCT data are focused on morphological changes during OTM [4, 14, 15]. However, few data are available regarding the correlation between incisor retraction and alveolar bone remodeling. To facilitate dentoalveolar bone risk assessment in the inter-disciplinary management of bimaxillary protrusion, we aimed to investigate changes in the alveolar bone in the incisor region before (T0) and after treatment (T1) and to explore its relationship with displacement of root apex as well as changes in the inclination of maxillary incisors. The null hypotheses of this study were that: (1) there was no changes in alveolar bone thickness (ABT), height (ABH), and area (ABA) before and after treatment; (2) the displacement of root apex and change in the inclination of maxillary incisor would not affect the alveolar bone area (ABA).

## Methods

### Materials

The study was designed as a retrospective cohort study. Ethical approval for the study was obtained from the Institutional Ethics Committee. The Institutional Review Board (IRB) number is 2019NL-064(KS). The study included data from 38 patients (31 females and 7 males), retrieved from the archive of medical school of university. All the patients started treatment from Jan 1st, 2014 to Dec 31st, 2017, and finished before June 30, 2019. The mean preoperative age was 19.52 years with an overall range of 15–33 years. Treatment time was 26 ± 4 months.

All patients were treated with interactive self-ligating brackets (American Orthodontics, USA). The self-ligating brackets are active in the incisors and passive in the canines, premolars, and molars with an MB prescription. The treatment protocol was briefly described as below. Alignment was achieved by sequential insertion of 0.014- and 0.018-in. nickel-titanium (NiTi) archwires, followed by levelling with 0.016 × 0.022- and 0.018 × 0.025-in. NiTi archwires, and space closure was finished with 0.019 × 0.025-in. stainless-steel (SS) archwires by en masse retraction and sliding mechanics. A retraction force of 100 g from the temporary anchorage devices (TADs) to the hook between lateral incisors and canines was utilized. The TADs were inserted at about 5 mm above the gingival margin between the 2nd premolars and 1st molars to achieve maximal anchorage. Appointment intervals were approximately 6 weeks. A lateral x-ray was used to perform the cephalometric analysis. The cephalometric data were presented in Table 1.

**Table 1:** The cephalometric data before and after orthodontic treatment

Parameter	T0					T1					*P* Value
	Mean	SD	25% Percentile	Median	75% Percentile	Mean	SD	25% Percentile	Median	75% Percentile	
SNA(°)	81.7	3.1	80.1	81.8	82.7	81.2	2.2	80.0	80.7	82.9	0.4^W^
SNB(°)	75.7	3.1	74.0	75.7	77.6	75.6	2.4	73.6	75.9	77.1	0.8^T^
ANB(°)	6.0	1.3	4.9	6.0	6.8	5.6	1.2	4.5	5.5	6.4	0.1^T^
U1-SN(°)	108.8	6.7	104.4	108.2	114.5	99.2	6.5	94.6	100.5	102.0	< 0.001^T^
U1-NA(°)	26.4	5.7	22.1	27.2	30.7	19.0	6.9	15.2	19.6	24.6	< 0.001^T^
U1-NA (mm)	2.4	0.8	1.8	2.4	3.0	1.6	0.7	1.0	1.5	2.0	< 0.001^T^
MP-SN(°)	39.3	5.7	35.2	39.5	42.7	38.8	5.2	36.1	38.5	41.7	0.6^T^
MP-FH(°)	30.0	5.7	25.3	29.5	32.8	30.3	4.8	26.1	30.3	33.6	0.4^W^
L1-MP(°)	98.5	6.4	93.8	98.6	104.5	92.1	7.5	84.7	93.7	97.0	< 0.01^T^
L1-NB(°)	34.1	5.9	31.3	34.2	38.3	27.3	6.6	23.3	87.1	32.0	< 0.001^T^
L1-NB (mm)	4.7	1.2	4.0	4.9	5.4	3.1	0.9	2.5	3.1	3.8	< 0.001^T^
U1-L1(°)	113.0	6.7	108.2	113.8	117.8	129.3	8.7	123.1	129.5	134.0	< 0.001^T^

*SD* standard deviation, *T* paired t-test, *W* Wilcoxon test

A sample size calculation was undertaken using the PASS software package (Version 15.0; NCSS, USA). Since no relevant data regarding the regression analysis between changes in ABA, changes in inclination of maxillary incisor, and displacement of tooth apex was reported, the sample size calculation was based on our pilot study (*n* = 8). The pilot study estimated that R^2^ was 0.63. Based on a significance level of alpha 0.05, the sample size was calculated to achieve an 80% power and the sample size calculation showed that 27 subjects were necessary.

Inclusion criteria for bimaxillary protrusion patients are:

1. With complete CBCT data before(T0) and after treatment(T1)
2. Class I canine and molar relationship;
3. Pretreatment interincisal angle less than 124° with a crowding of less than 4 mm in the maxillary arch;
4. Full permanent dentition anterior to the first molars;
5. A minimum age of 15 in girls and 18 in boys to reduce effects of growth on dental structures.

Patients with previous orthodontic treatment, cleft lip palate, impacted anterior teeth, congenital tooth loss except third molars, systemic diseases and compromised periodontium were excluded.

### 3D image processing and measurements

All pretreatment and posttreatment CBCT were taken by the same machine. CBCT scans (NewTomVG, Quantitative Radiology, Verona, Italy) were taken before (T0) and after treatment (T1). The following imaging acquisition parameters were used: 16 × 16 cm field of view (FOV), 5 mA, 110 kV, and 3.6 s exposure time, which generated an isotropic voxel size of 0.3 mm. The effective dose of radiation was approximately 80 μSv.

The examined subjects were positioned in the sagittal plane perpendicular to the floor, which was parallel to the Frankfort plane. All CBCT data were exported to digital imaging and communications in medicine (DICOM) format. The 3D images were reconstructed using NNT Viewer software (NewTomVG, Quantitative Radiology, Verona, Italy).

**Fig. 1:**
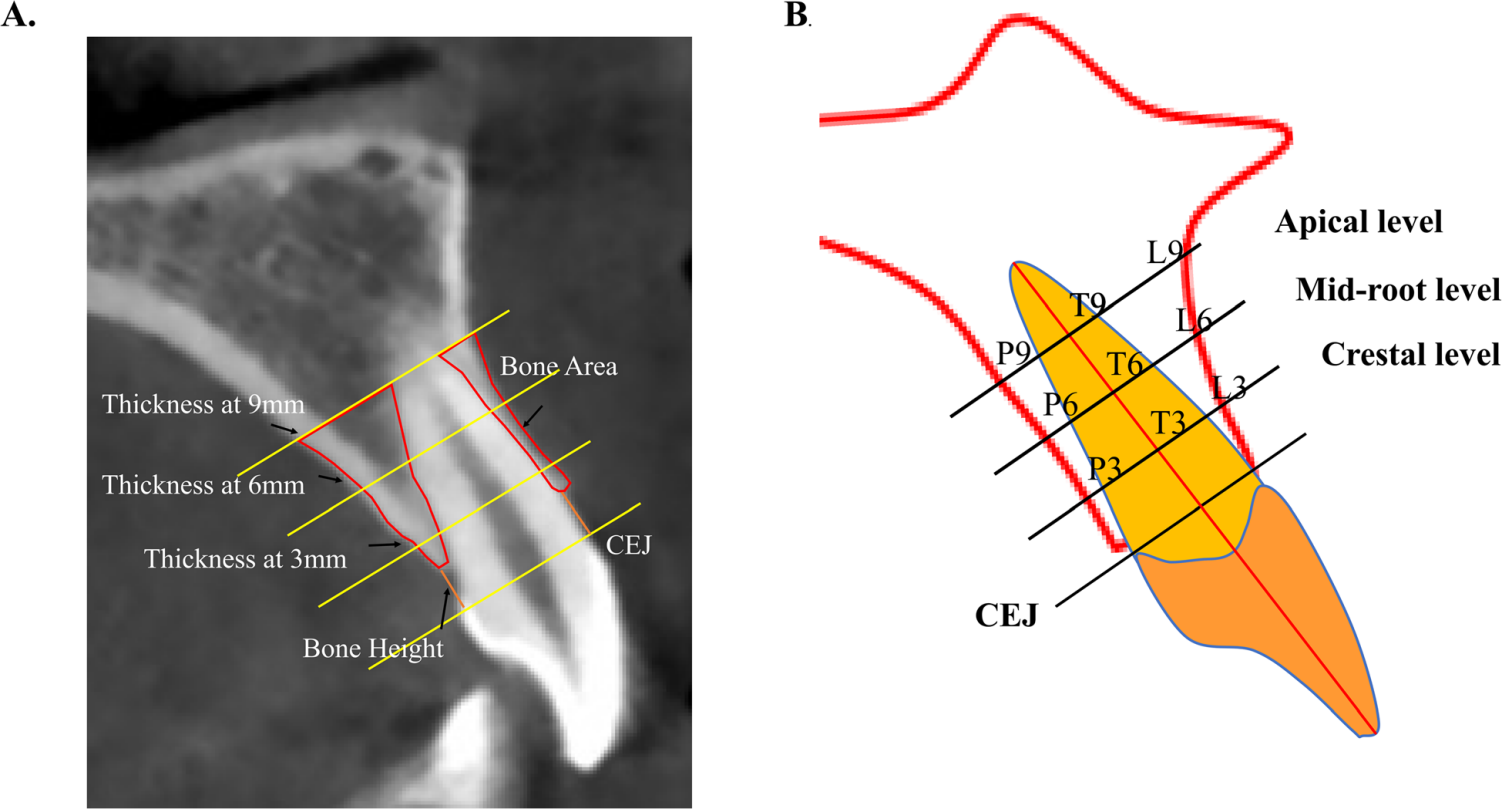
Determination of alveolar bone morphology in incisors. Alveolar bone thickness was measured at the apical, mid-root and crestal level. CEJ: cementoenamel junction

In NNT Viewer, the sagittal slices were cut through the center of apical foramen and parallel to the long axis of individual incisors. These images were imported into the software ImageJ (version 2.0, NIH, Bethesda, Md), within which the following variables were measured: (1) alveolar bone thickness (ABT) on the buccal and palatal side at 3, 6 and 9 mm from the cementoenamel junction (CEJ), which was designated as crestal, mid-root and apical third; (2) labial and palatal alveolar bone area (ABA); (3) labial and palatal alveolar bone height (ABH), defined as the distance from the CEJ to the alveolar ridge crest (Fig. 1 a and b).

Tooth movement of individual maxillary incisor was determined by apex displacement and axis inclination alteration (modified from Kyoung-Won et al.) [16]. The apical displacement was registered as the changes of the distance between the upper incisors’ apical foramen and the palatal cortical bone, with apex moving towards buccal plates defined as positive and towards palatal plates as negative. The rationale of the reference mark is that the palatal cortical plate does not undergo structural changes and remain to be stable in adult patients [17]. Moreover, this reference mark was very close to the investigated area avoiding unwanted changes of remote structures and minimizing errors of measurement. Inclination of individual incisor was determined as the lower backward angle between the long axis and Frankfort Horizontal (FH) plane.

### Statistical analyses

All measurements were conducted by one trained examiner. To reduce the measurement error, we took the average value of three measurements whose time interval was one-month as the result. The inter-examiner agreement was performed by another experienced investigator. Repeated measurements were examined by the paired t-test (systematic errors) and the Dahlberg formula (casual errors) [16]. No significant systematic errors were found (*P* > 0.1), and the random errors were small, showing high rates of reproducibility. The normality of distribution of the variables was assessed by Shapiro-Wilks test. Interphase changes (T1–T0) were calculated, and if normally distributed, these were compared using paired t-tests; if this was not the case, the Wilcoxon test was used. Pearson’s Chi-square test was applied to determine whether different ways of teeth movement affected the measurement results before and after treatment. Regression analysis was performed to determine the relationship between changes in the alveolar bone area and apex displacement as well as changes in the inclination of incisors. All statistical analyses were performed with GraphPad Prism 8.0.1 and SPSS Statistics with a significance level of 0.05.

## Results

### Incisor tooth movement

Inclination of incisors related to FH plane and anatomic position of apex foramen to palatal cortical plate was determined to reflect the 3-dimensional position of the incisors. Although we selected cases with a crowding less than 4 mm, the inclination of central and lateral incisors demonstrated a rather large discrepancy, a result of crowding and irregularity in the maxillary front region. The inclination of central and lateral was significantly reduced after treatment (Table 2). In addition, the distance of apex of central and lateral incisors to palatal cortical plate was significantly reduced with an average of 1.0 mm and 1.9 respectively, showing that root apex was moved towards the palatal cortical plate (Table 2).

**Table 2:** Changes in the root position during orthodontic tooth movement (Mean ± SD)

		T0	T1	T1-T0	*P* Value
Central	Inclination to FH plane(°)	119.4 ± 7.7	111.9 ± 9.2	−7.5 ± 10.6	0.03^T^
	Distance from apex to palatal cortical plate (mm)	6.4 ± 1.8	5.4 ± 3.0	−1.0 ± 2.2	0.02^T^
Lateral	Inclination to FH plane(°)	117.0 ± 7.7	113.3 ± 7.3	−3.7 ± 9.9	0.04^T^
	Distance from apex to palatal cortical plate (mm)	5.8 ± 1.7	3.9 ± 2.2	−1.9 ± 2.2	0.02^T^

*SD* standard deviation, *T* paired t-test

### Alveolar bone thickness

We next explored changes of the buccal, palatal and total ABT at the crestal, mid-root and apical third. The labial ABT of both central and lateral incisors at the mid-root third was increased significantly (*P* < 0.05), while no significant difference was observed at the crestal and apical third. In contrast, the palatal ABT of the central and lateral incisors at all three levels were consistently decreased (*P* < 0.05). Regarding changes in the total ABT, significant reduction was observed in both central and lateral incisors at the crestal level, and in lateral incisors at the mid-root third level as well as apical third level (*P* < 0.05) (Table 3).

**Table 3:** Changes in alveolar bone thickness at the crestal, mid-root and apical third

		T0					T1					*P* Value
		Mean	SD	25% Percentile	Median	75% Percentile	Mean	SD	25% Percentile	Median	75% Percentile	
Central	Crestal- labial	0.8	0.3	0.7	0.8	1.0	0.8	0.3	0.6	0.8	1.0	0.2^T^
	Mid- labial	0.7	0.2	0.6	0.7	0.8	0.9	0.4	0.6	0.8	1.1	< 0.01^W^
	Apical- labial	0.9	0.3	0.7	0.9	1.0	1.0	0.6	0.7	0.9	1.3	0.3^W^
	Crestal-palatal	1.6	0.4	1.3	1.5	1.9	0.7	0.9	0.0	0.4	1.3	< 0.001^W^
	Mid- palatal	2.9	0.8	2.2	2.8	3.4	2.2	1.4	1.3	2.0	3.3	< 0.001^T^
	Apical- palatal	4.4	1.4	3.5	4.4	5.2	4.2	1.8	3.0	4.1	5.6	0.04^W^
	Crestal- total	8.3	0.7	7.8	8.1	8.6	7.9	1.1	7.3	7.7	8.4	< 0.001^T^
	Mid- total	8.6	0.9	7.8	8.4	9.1	8.5	1.5	7.4	8.3	9.7	0.6^T^
	Apical- total	8.8	1.1	8.0	8.7	9.3	9.0	1.7	7.6	9.1	10.0	0.4^W^
Lateral	Crestal- labial	0.7	0.5	0.4	0.7	0.9	0.6	0.4	0.5	0.7	0.8	0.3^W^
	Mid- labial	0.5	0.3	0.2	0.5	0.7	0.6	0.4	0.4	0.7	0.9	< 0.001^T^
	Apical- labial	0.5	0.5	0.0	0.5	0.8	0.7	0.5	0.3	0.7	1.0	0.1^W^
	Crestal-palatal	1.4	0.5	1.0	1.3	1.8	0.4	0.6	0.0	0.0	0.8	< 0.001^T^
	Mid- palatal	2.5	0.9	1.9	2.3	2.9	1.5	1.1	0.1	1.4	2.3	< 0.001^W^
	Apical- palatal	3.8	1.3	3.1	3.6	4.6	3.1	1.6	2.0	2.8	4.2	< 0.001^W^
	Crestal- total	7.9	0.8	7.5	7.9	8.4	7.1	0.9	6.3	6.9	7.6	< 0.001^T^
	Mid- total	8.1	1.2	7.4	7.8	8.9	7.5	1.2	6.5	7.2	8.5	< 0.001^T^
	Apical- total	8.2	1.3	7.1	8.2	9.0	7.7	1.5	6.7	7.4	8.9	< 0.01^T^

*SD* standard deviation, *T* paired t-test, *W* Wilcoxon test

### Alveolar bone height

In consistence with the reduced ABA, a significant decrease in the palatal ABH of both central and lateral incisors was observed between T0 and T1 (*P* < 0.001), indicating palatal alveolar bone resorption. However, in terms of labial ABH, no significant difference was observed (Table 4).

**Table 4:** Changes in the labial and palatal alveolar bone height in central incisors and lateral incisors before (T0) and After treatment (T1)

		T0					T1					
		Mean	SD	25% Percentile	Median	75% Percentile	Mean	SD	25% Percentile	Median	75% Percentile	*P* Value
Central incisor	Labial	1.6	0.6	1.1	1.4	1.8	1.7	0.7	1.3	1.5	1.9	0.1^W^
	Palatal	1.4	0.5	1.1	1.3	1.5	3.4	2.0	1.6	2.9	4.4	< 0.001^W^
Lateral incisor	Labial	2.0	0.8	1.4	1.8	2.2	2.1	1.5	1.4	1.7	2.3	0.9^W^
	Palatal	1.5	0.6	1.1	1.4	1.8	4.1	1.8	2.6	4.3	5.3	< 0.001^W^

*W* Wilcoxon test

### Alveolar bone area

**Fig. 2:**
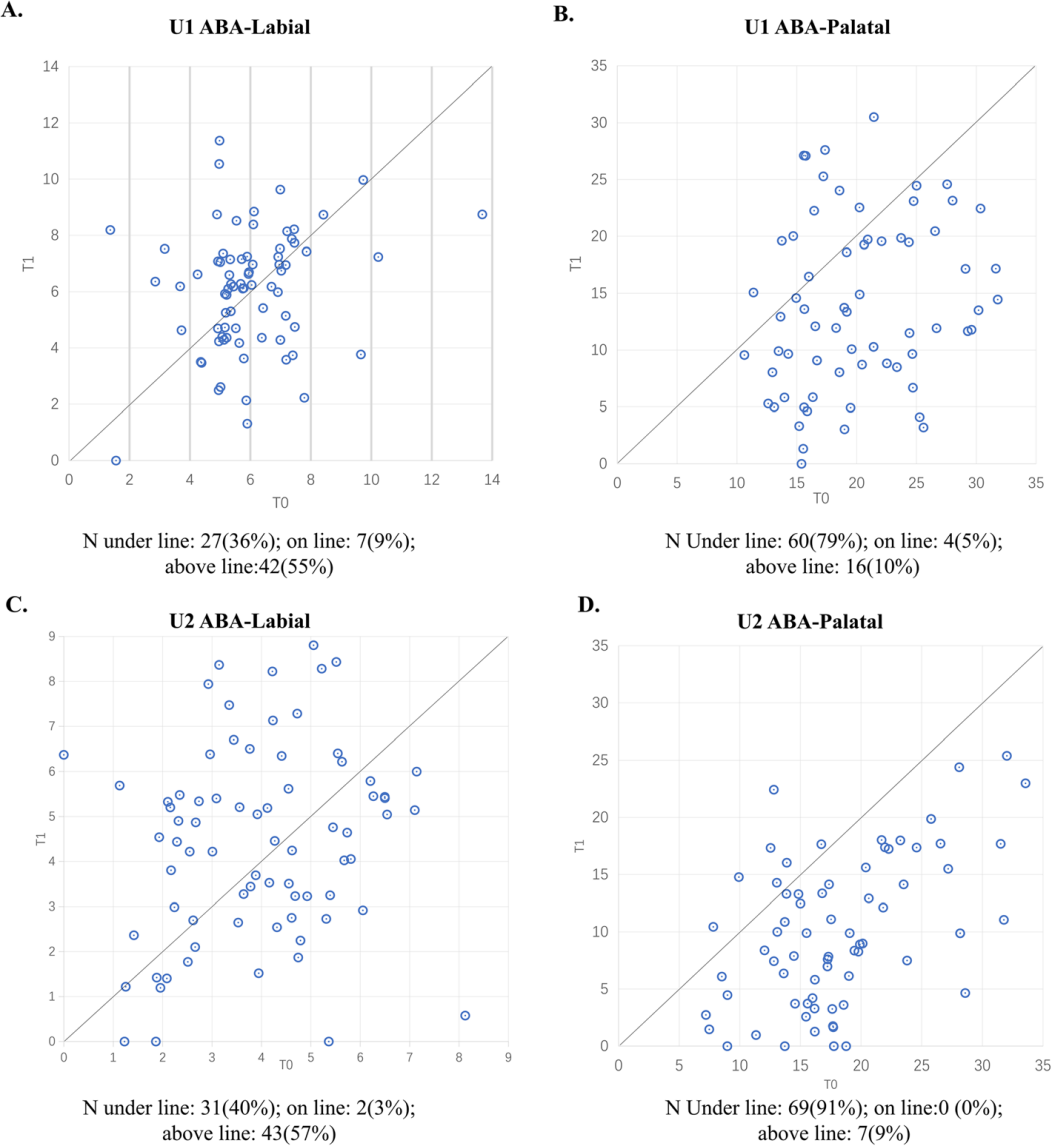
Scatterplots showing the changes in alveolar bone area at T0 and T1

Decreased palatal ABA was observed in the majority of samples (74% in central incisors and 86% in lateral incisors). In contrast, the labial ABA was decreased in 36% of central incisors and 40% of lateral incisors respectively, while it was increased in 55% of central incisors and 57% of lateral incisors. An average bone loss of 20.5 and 40.0% on the palatal side was observed in the central and lateral incisors respectively. Further statistical paired t-tests revealed that reduction in the palatal ABA of the central and lateral incisors was statistically significant (*P* < 0.001), while no difference was observed in the labial side (*P* > 0.05) (Fig. 2, Table 5).

**Fig. 3:**
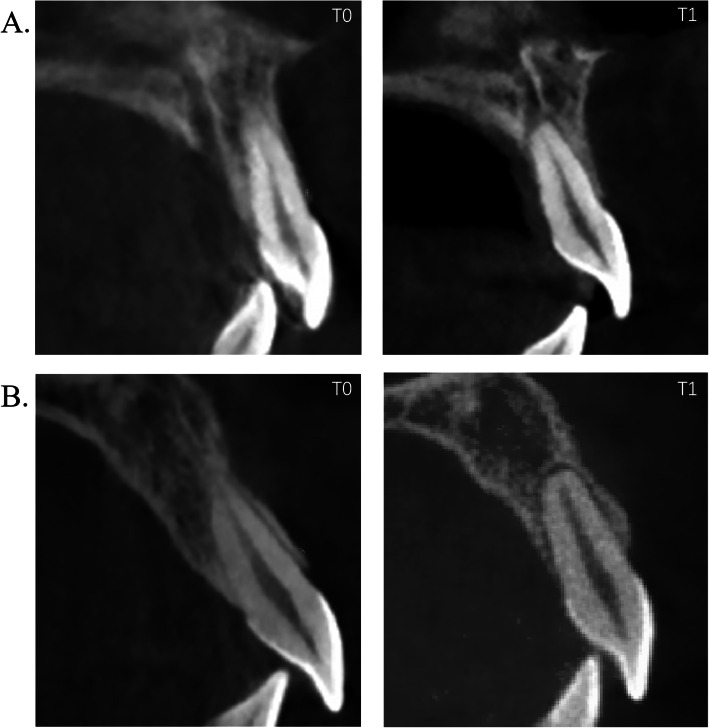
Characteristic changes in the alveolar bone during incisor retraction. **a**. Typical alteration in the alveolar bone at the central incisor with a massive palatal apex displacement, i.e. bodily movement; **b**. Typical alveolar bone remodeling at the central incisor with a large change in the inclination, i.e. tipping

**Table 5:** Changes in the labial and palatal alveolar bone area in central and lateral incisors before (T0) and after treatment (T1)

		T0					T1					
		Mean	SD	25% Percentile	Median	75% Percentile	Mean	SD	25% Percentile	Median	75% Percentile	*P* Value
Central incisor	Labial	6.2	2.3	5.0	5.8	7.0	6.1	2.3	4.4	6.3	7.4	0.9^W^
	Palatal	20.3	5.3	15.7	19.3	24.7	16.1	11.2	8.7	13.5	20.3	< 0.001^W^
Lateral incisor	Labial	4.0	2.2	2.6	3.7	5.1	4.6	2.2	2.9	4.7	6.2	0.1^W^
	Palatal	18.0	5.9	13.9	17.2	21.4	10.5	7.6	4.3	9.4	15.3	< 0.001^W^

*W* Wilcoxon test

### Relationship between changes of labial ABA and tooth movement types

Among all 152 teeth, the apex of 38 (25%) teeth was moved toward labial side, while the remaining 114 (75%) was toward palatal side. In the meantime, the inclination of 26 (17.1%) teeth increased, whereas the remaining 126 (82.9%) decreased.

Four different ways of tooth movement were achieved through permutation and combination theory: labial apex displacement (+) & inclination increase (+), labial apex displacement (+) & inclination decrease (−), palatal apex displacement (−) & inclination increase (+), and palatal apex displacement (−) & inclination decrease (−). Among them, there was no labial apex displacement (+) & inclination increase (+) in the subjects. Pearson’s Chi-square test was applied to determine whether three ways of tooth movement affected the labial ABA before and after treatment, which showed a strong correlation between labial ABA changes and tooth movement type (*P* < 0.001) (Table 6). Labially-displaced apex was correlated with decreased labial ABA, while palatally-displaced apex was correlated with increased labial ABA.

**Table 6:** Frequency of labial alveolar bone area (ABA) increase and decrease in upper four incisors among different ways of tooth movement using a Pearson X^2^ Test (*n* = 152)

Apex displacement	Inclination	Labial ABA Increase (+)	Labial ABA Decrease (−)	X^2^	*P* Value
Labial (+)	Decrease (−)	9	29	33.1	< 0.001
Palatal (−)	Increase (+)	23	3		
Palatal (−)	Decrease (−)	61	27		

### Relationship between changes of palatal ABA and tooth movement types

As a reduction in palatal ABA was observed in most patients (Fig. 3), a multivariate linear regression analysis was used to further explore the relationship between alterations of palatal ABA and tooth movement type, showing that the changes (T1-T0) of palatal ABA were remarkably correlated with the apex displacement and changes of inclination (T1-T0) before and after treatment (F = 107.9, *P* < 0.001) (Table 7). This model can be also described by using this equation: Changes of palatal ABA (T1-T0) = − 3.3- 0.1× changes of inclination (T1-T0) + 2.5× apex displacement (T1-T0) (Table 7).

**Table 7:** Multivariate linear regression analysis of apex displacement and changes in inclination (T1-T0) for the changes of palatal alveolar bone area (ABA) (T1-T0) in maxillary incisors

dependent variable	constant and influencing factors	R^2^	F value	b (SE)	*P* value
Changes in palatal ABA(T1-T0)	Constant	0.6	108.0	−3.3 (0.8)	< 0.001
	Changes in Inclination(T1-T0)			−0.1 (0.1)	0.02
	Apex Displacement			2.5 (0.3)	< 0.001

The equation can be used to predict changes of palatal ABA with certain values of the predictors. For example, the predicted changes of palatal ABA for a patient with palatal apex displacement of − 3 mm and decrease of 8° in inclination is − 3.3-0.1× (− 8) + 2.5× (− 3), which equals to − 7.5 mm^2^.

## Discussion

Periodontal health is of great concern for the orthodontists and periodontists, especially in the inter-disciplinary management of bimaxillary protrusion patients [18]. Since protrusive lips is one chief complaint of patients seeking orthodontic treatment in the Eastern Asian community, four bicuspid extraction was commonly utilized to correct dental and lip protrusion [2]. The principal finding of the present research was that mass retraction of maxillary incisors leaded to a significant reduction in the ABA on the palatal side, and apex displacement is the major factor that contributed to decreased palatal ABA. Moreover, multiple line regression indicated an equation to potentially predicate alveolar bone resorption on the palatal side by integrating apex displacement and changes in the inclination of incisors. Therefore, the null hypotheses were rejected.

Dentists have been seeking personalized approaches to improve treatment outcome and avoid deleterious sequelae for a long time [19]. The new era of precision medicine requires a personalized, or individualized treatment plan and case management [20]. The use of digital orthodontic setups has grown quickly to aid individualized plan, mimic tooth movement and predict treatment outcome [21]. Three-dimensional CBCT imaging have exponentially enhanced the capability to evaluate regional anatomy in the alveolar process and assess periodontal risks during tooth movement [7].

Alveolar bone, which originates from the dental follicle during embryogenesis, is unique in its dynamic remodeling capacity during tooth eruption and OTM [7]. It has been long accepted that OTM is a dynamic process whereby the application of orthodontic force induces bone resorption on the pressure side and bone apposition on the tension side, which maintains structural integrity of the alveolar bone [8]. Although the alveolar bone might be dynamically remodeled to house moving teeth in growing adolescents, OTM is limited by the cortical plates of the alveolus at the level of the incisor apices, which can be regarded as “orthodontic walls”, in non-growing adults [5]. Moving teeth out of these boundaries may lead to occurrence of severe iatrogenic sequelae of alveolar bone resorption, and this is especially disastrous in adult patients [5, 22]. The width of the anterior palate at the level of the apex remained unaltered despite long-term incisor retraction in adult patients [23]. Indeed, we observed significant decrease in the height, thickness and area of alveolar bone on the palatal side.

The acumen of CBCT may help orthodontists and periodontists assess periodontal bone status and remodeling in OTM [7]. Stages of alignment, bite opening and space closing are integrated processes in extraction cases. It has been demonstrated that alignment of anterior teeth by tipping leaded to significant vertical and horizontal loss of alveolar bone in non-extraction cases [24–26]. In addition, Yodthong et al. investigated the effect of incisor retraction on changes of alveolar bone. CBCT was taken before retraction and after 6 months of retraction. They reported an increase of 0.4 mm in ABT at the crestal level on the labial side, 0.2 mm and 0.6 mm decrease at crestal and apical level on the palatal side respectively for the central incisors, indicating that incisor retraction is a risk of alveolar bone resorption in bimaxillary protrusion patients. However, the total thickness of the alveolar bone even increased an average of 0.6 mm at the apical level [27]. Sarikaya et al. reported that no changes in the labial side were observed, while decrease in ABT in the maxillary arch was observed in four premolar extraction cases with 0.7 mm and 1.2 mm at the crestal and mid-root level [4]. Our results were consistent with Ahn et al. that remarkable bone absorption can be found in all three levels on the palatal side, while bone thickness on the labial side increased in the middle third, by 0.27 mm for upper central incisors and by 0.65 mm for lateral incisors, with statistically significant differences [14].

In the process of assessing the periodontal risk, dentists should not only evaluate anatomic features of alveolar bone before treatment, but also the remodeling potential and 3-dimensional position of targeted teeth in the alveolus [28]. 3D imaging techniques may help orthodontic-periodontal interdisciplinary coordination in managing periodontal iatrogenic effects [29]. Consistent absorption of alveolar bone on the palatal side in our study and in Ahn et al. [14] indicates that retraction actually moves incisors “through-the-bone”. Despite the increase in alveolar bone thickness and area on the buccal side, decrease in the total thickness and area in the incisor region suggests that orthodontic treatment may lead to increased risks for periodontal sequelae.

Digital setups have been widely used for diagnosis, treatment planning, indirect bonding, simulating treatment, and designing and producing orthodontic appliances, especially in the aligner techniques [21, 30, 31]. Much emphasis has been laid on crown position rather than root position because spatial position of roots is not available until the emergence of CBCT imaging and roots are usually not directly related to esthetics and occlusal contact [32]. However, if the alveolar bone is not taken into consideration, such digital setup may push the tooth out of the bone for considerable distance. Our present data suggest that without taking the alveolar bone remodeling into consideration, 3D Digital setups, especially in aligners, may push the teeth out of the bone boundary.

Both the height and thickness of alveolar bone are critical factors to protect the teeth from plaque-induced (i.e., periodontitis) and non-plaque-induced gingival lesions [33]. Thinner alveolar wall after incisor retraction may lead to dehiscences or fenestrations that compromise alveolar bone support [17]. In order to investigate overall effect of incisor retraction on alveolar remodeling, we made a preliminary attempt to describe changes in alveolar bone area, which reflected changes in ABL and ABT; we found that apical displacement and alterations in inclination were significantly related to ABA on the palatal side; moreover, we developed an equation to describe changes in ABA, which described that changes of palatal ABA (T1-T0) is equal to (− 3.258–0.139× changes of inclination (T1-T0) + 2.533 × apex displacement (T1-T0)).

We utilized changes in the inclination of each individual incisor rather than measurements from cephalometric to generate an equation for prediction of individual palatal ABA. Such equation may customize both periodontal and orthodontic consideration in adult patients, especially determining whether bone augmentation surgery was needed to aid orthodontic therapy, commonly recognized as surgically facilitated orthodontic therapy [SFOT] [34] or periodontally accelerated osteogenic orthodontics [PAOO]) [35]. Our present equation further supports the conception that bodily retraction would lead to proximity of root apex to palatal cortical plate and extensive alveolar bone resorption [36], and this jeopardy is particularly pertinent in adults as numbers and regeneration capability of osteoblasts in the periosteum reduces with aging [37]. Therefore, clinicians should not design bodily movement of a large distance to avoid placing the teeth out of the bone boundary during treatment planning.

Many factors may influence OTM and alveolar bone remodeling in the periodontium, such as force magnitude (light or heavy), force type (intermittent, interrupted or continuous), age(adolescents or adults), distance of movement, type of OTM (tipping, controlled tipping, translation, intrusion, extrusion), speed of space closure [27]. For example, intrusion of incisors into a wider alveolus may increase alveolar bone support, thereby compensating the palatal bone resorption [38]; however, excessive intrusion may lead to remarkable root resorption [39]. Cortical plates at the root apex level did not remodel in adult patients [5]; therefore, it may be utilized as a reliable mark to reflect the position of incisors. In our present study, we did not measure the distance of retraction for individual teeth, because the distance of root apex to palatal plates and inclination of incisors actually reflected changes in the position of incisors. Another issue was that we did not include the vertical position of root apex. Only minimal intrusion of incisors can be observed during retraction of incisors in bimaxillary protrusion patients [27], and large quantities of incisor intrusion can only be observed in class II patients, especially division 2 type with reverse curve of Spee [40]. Data from the 2-dimensional changes in the lateral cephalograms were most often utilized to represent overall intrusion [27, 40, 41]; therefore, we did not further investigate influence of intrusion of incisor in 2-dimensional cephalometric on ABA.

We utilized adult patients to minimize the influence of growth on alveolar bone remodeling; therefore, interpretation of data in adolescents should be cautious. A key question critical in alveolar bone remodeling during OTM is elasticity and flexibility of alveolar bone, which undergoes rapid remodeling with low mineralization and stiffness even in adults [42]. More CBCT data, especially from growing subjects, are needed to better predict changes in the periodontium and optimize periodontal risk assessment [25]. A pre- and post-CBCT may be taken in cases who need mass incisor retraction, in order to assess potential periodontal risks such as fenestrations and dehiscence and provide individualized suggestion for long term periodontal maintenance for each subject after treatment. Clinicians should not be blinded to the periodontal sequalae induced by mass retraction, although soft tissue may hide severe bone resorption, fenestration and dehiscence on the palatal side [43]. Moreover, patients should be informed of their periodontal situation, since such severe bone loss may be disastrous once the patient lose the teeth due to trauma or periapical diseases [44].

Currently, data regarding long-term alveolar bone remodeling after retention is not available except one case report [45]. In the case report, significant alveolar bone formation was observed on the palatal side of the maxillary incisors 10 years after retention [45]. Long-term studies are needed to confirm the alveolar bone apposition on the palatal side, and to improve the predication of alveolar bone remodeling in our equation.

Although CBCT may reduce periodontal risks in OTM, ALARA (As Low As Reasonably Achievable) principle should be adhered to, especially in growing adolescents. Each subject should be evaluated individually based on their unique treatment needs and set of circumstances [7]. Digital set-up provides dentists unprecedented opportunities to predict the final position of the crowns before the treatment [46]. Our present research indicated that with the help of CBCT imaging, dentists may further predict the final root position in the alveolus in the future.

The accuracy of CBCT imaging is closely related to voxel size. A smaller voxel size such as 0.2 mm provides a superior accuracy compared to a size of 0.4 mm in assessing the alveolar bone and soft tissues [47]. However, the patients are exposed to a higher dose of ionizing radiation under the condition of a smaller voxel size [26]. Indeed, a FOV of 16 × 16 cm and a voxel size of 0.3 mm used in this study may compromise the accuracy in assessing the alveolar bone. The approximate effective dose of 80 μSv in our present study, which was similar to the study of Pauwels et al. [48], may reduce the radiation risks under a lower voxel size.

We utilized the predominant vision-based method to discriminate dental structures in the dental literatures. Such vision-based method has been widely adopted to study the alveolar bone [47], temporomandibular joint [49], and airway space [50], and it is time-efficient. However, conventional vision-based method may be less accurate and reproducible when compared to gray value–assisted method proposed [10].

We only included adult cases in the present study, which partially explain the small sample size. Further long-term prospective studies including follow-up observation and studies in adolescents may bring us more information on the periodontal remodeling during orthodontic treatment.

## Conclusions

1. Retraction of incisors in bimaxillary protrusion patients leaded to significant bone resorption on the palatal side, which may compromise periodontal bone support;
2. Palatal displacement of root apex was highly correlated with palatal bone resorption;
3. The area of bone resorption might be predicted by an equation that incorporated the displacement of root apex and changes in the incisor inclination;
4. The equation in predicating area of alveolar bone may reduce the risks of placing the teeth out of the bone boundary during 3D digital setups.

## Data Availability

The raw data in this study are available by contacting corresponding author Dr. Lang Lei, China-Email address: leilangdental@163.com.

## Abbreviations

CBCT: Cone-beam computed tomography
ABT: Alveolar bone thickness
ABH: Alveolar bone height
ABA: Alveolar bone area
OTM: Orthodontic tooth movement
SWA: Straight wire appliances
TADs: Temporary anchorage devices
CEJ: Cementoenamel junction
FH plane: Frankfort horizontal plane
